# Synthesis of biotinylated probes of artemisinin for affinity labeling

**DOI:** 10.1016/j.dib.2015.04.017

**Published:** 2015-05-05

**Authors:** Benetode Konziase

**Affiliations:** Graduate School of Pharmaceutical Sciences, Osaka University, 1-6 Yamada-oka, Suita, Osaka 565-0871, Japan

**Keywords:** *m*/*z*, mass-to-charge ratio, DPPA, diphenylphosphoryl azide, Et_2_O, diethyl ether, EtOAc, ethyl acetate, Et_3_N, triethyl amine, MeOH, methanol, NaOMe, sodium methoxide, THF, tetrahydrofuran, Synthesis, Biotinylated probes, Artemisinin, Affinity labeling

## Abstract

In this data article, we described the synthetic routes to four biotinylated probes (**2**, **3**, **4**, and **5**) of artemisinin and the associated experimental procedures. We also provided the physical data for the synthesized compounds. These synthesized biotinylated probes of artemisinin are useful molecular tools for the affinity-labeling study of target receptor proteins of artemisinin in tropical pathogens such as *Trypanosoma, Leishmania, and Schistosoma.* The data provided herein are related to “Biotinylated probes of artemisinin with labeling affinity toward *Trypanosoma brucei brucei* target proteins”, by Konziase (Anal. Biochem. (2015)).

**Specifications table**

**Table:** 

Subject area	Organic chemistry
More specific subject area	Organic synthesis
Type of data	Synthetic schemes, experimental procedures, physical data
How data was acquired	Chemical reactions; normal phase column chromatography; NMR spectroscopy: JNM-GX-500 (JEOL), Lambda 500 (JEOL), Inova 600 (Varian); mass spectroscopy: JMS SX-102 (JEOL); IR spectroscopy: FT-IR-5300 (JASCO); polarimetry: DIP-370 (JASCO)
Data format	Analyzed, text, schemes
Experimental factors	N/A
Experimental features	Chemical reactions were performed under argon gas unless otherwise indicated; the diazirine-containing probes were synthesized in brown opaque chemical flasks or transparent chemical flasks wrapped with aluminium foil due to photosensitivity.
Data source location	Osaka, Japan
Data accessibility	Data are available with this article

**Value of the data**

• To reproduce all the experiments described in the research article ref [1].
• To detect and isolate trypanosomal candidate target proteins of artemisinin.
• To study the target receptors of artemisinin in *Leishmania* or *Schistosoma*.

## 1 Materials and methods

### 1.1 General

^1^H-NMR and ^13^C-NMR spectra in CDCl_3_ or CD_3_OD with TMS as the internal standard were recorded using a JNM-GX-500 or Lambda 500 (JEOL, Tokyo, Japan) NMR spectrometer operating at 500 MHz and 125 MHz, respectively. 2D NMR data in CDCl_3_ were recorded using a Varian Inova 600 (Varian, Tokyo, Japan) NMR spectrometer operating at 600 MHz. Chemical shifts (δ) were reported in parts per million (ppm) and the multiplicities were designated as follows: s (singlet), d (doublet), t (triplet), m (multiplet), dd (doublet of doublets), ddd (doublet of doublet of doublets), brd (broad doublet), t-like (triplet like), dt (doublet of triplets). The coupling constants (*J*) were reported in Hz. Fast atom bombardment (FAB) and high-resolution fast atom bombardment (HR-FAB) mass spectra were recorded with a JMS SX-102 (JEOL, Tokyo, Japan) spectrometer in positive ion mode using magic bullet (5:1 dithiothreitol/dithioerythritol; Tokyo Kasei Kogyo) or *m*-nitrobenzyl alcohol as the matrix. Infrared (IR) spectra were recorded by a diffusion-reflection method on KBr powder using an FT-IR-5300 (JASCO, Tokyo, Japan) spectrometer. Shoulder bands in the IR spectra were designated by sh. Optical rotations were measured in a 0.5 dm length cell with a DIP-370 (JASCO, Tokyo, Japan) digital polarimeter. For column chromatography, silica gel (Fuji Sylisia BW-200 or Merck 60–230 mesh) and octadecyl silane ODS (Cosmosil 75C_18_ OPN, Nacalai-Tesque) were used. Chemical reactions were performed under Ar gas unless otherwise indicated. TLC analyses were performed using normal-phase pre-coated plates (Kiesel gel 60F_254_, Merck) and reversed-phase high-performance thin-layer chromatography (HPTLC) plates (RP-18 WF_254S_, Merck). The spots on the thin-layer chromatograms were detected under UV light at 254 and 366 nm and visualized with either *p*-anisaldehyde/H_2_SO_4_ (5 mL of AcOH, 25 mL of *c*-H_2_SO_4_, 425 mL of EtOH, and 25 mL of water) or phosphomolybdic acid (5 g in 100 mL of EtOH) spraying reagents and subsequent heating.

### 1.2 Synthetic methods

By following the synthetic routes described here below, we successfully synthesized four biotinylated probes of artemisinin that were used as molecular tools for the affinity labeling of *Trypanosoma brucei brucei* target proteins [1].

#### 1.2.1 Synthetic route to the biotinylated photoaffinity probe **2**

**Scheme 1:**
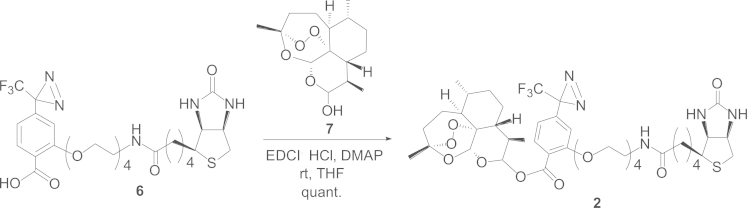
Synthetic route to the biotinylated photoaffinity probe **2**.

We first prepared the photoaffinity-labeling unit **6** according to the method described by Hatanaka et al. [2,3]. Direct condensation of **6** with dihydroartemisinin (**7**) (purchased from Tokyo Chemical Industry, Japan) using 1-[3-(dimethylamino)propyl]-3-ethylcarbodiimide hydrochloride (EDCI·HCl) and 4-(dimethylamino)pyridine (DMAP) in tetrahydrofuran afforded quantitatively probe **2** (Scheme 1).

*Preparation of probe **2**:* To a solution of **6** (26 mg, 0.043 mmol) in THF-CH_3_CN (1:1, 1.6 mL), were added dihydroartemisinin (**7**) (24.5 mg, 0.086 mmol), EDCI·HCl (24.8 mg, 3 mol equiv. to **6**), and DMAP (2.64 mg, 0.5 mol equiv. to **6**). The whole mixture was stirred at room temperature for 36 h. A crude mixture (108 mg) was obtained and applied to SiO_2_ column chromatography (CHCl_3_:CH_3_OH:H_2_O=7:3:1) yielding **2** (26 mg) quantitatively (Scheme 1).

Compound **2**: a white powder, [α]_D_^25^ 162.4 (*c* 0.43, CHCl_3_), IR (KBr): 2926, 1740, 1695, 1610 cm^−1^, ^1^H NMR (CDCl_3_) δ: 7.92 (1*H*, d, *J*=7.9 Hz, 6″-H), 6.81 (1*H*, d, *J*=7.9 Hz, 5″-H), 6.71 (1*H*, s, 3″-H), 5.96 (0.6*H*, d, *J*=9.7 Hz, 12β-H), 5.86 (0.4*H*, s, 12α-H), 5.49 (0.6*H*, s, 5-Ha), 5.43 (0.5*H*, s, 5-Hb), 4.48 (1*H*, m, 2′-H), 4.30 (1*H*, m, 3′-H), 4.19 (2*H*, t, *J*=4.9 Hz, 7″-H), 3.91 (2*H*, t, *J*=4.6 Hz, 14″-H), 3.42–3.76 (12*H*, m, 8″, 9″, 10″, 11″, 12″, 13″-H), 3.13 (1*H*, m, 1′-H), 2.88 (1*H*, dd, *J*=12.8, 5.5 Hz, 4′-Ha), 2.75 (1*H*, d, *J*=12.8 Hz, 4′-Hb), 2.55 (0.4*H*, m, 11-Hb), 2.37 (1*H*, ddd, *J*=4.3, 14.7, 14.7 Hz, 3α-H), 2.32 (2.6*H*, m, 11-Ha, 8′-H), 2.02 (1*H*, brd, *J*=*ca.* 14.5 Hz, 3β-H), 1.43 (3*H*, s, 14-H), 0.91–0.98 (6*H*, m, 13, 15-H). FAB-MS *m/z*: 892 [M+Na]^+^.

#### 1.2.2 Synthetic route to the biotinylated photoaffinity probe **3**

**Scheme 2:**
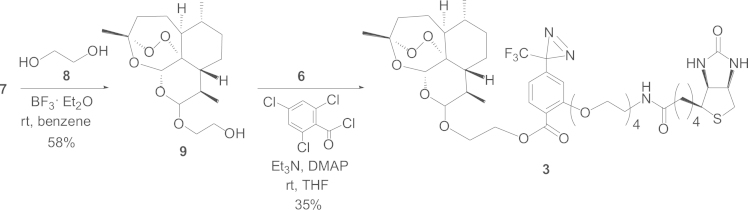
Synthetic route to the biotinylated photoaffinity probe **3**.

Dihydroartemisinin (**7**) was condensed to ethylene glycol (**8**) under boron trifluoride-etherated catalysis in benzene, affording **9**. Condensation of **9** with **6** *via* the Yamaguchi reaction afforded the probe **3** in a 35% yield (Scheme 2).

*Preparation of **9** from **7**:* To a solution of **7** (200 mg, 0.704 mmol) in benzene (10 mL), were added ethylene glycol (**8**) (800 μL, 888 mg) (purchased from Wako, Japan) and 5% v/v BF_3_·Et_2_O in benzene (400 μL). The mixture was stirred for 2 h. Then, 5% v/v BF_3_·Et_2_O in benzene (400 μL) was added again, and an additional stirring of the mixture was performed for 3 h. The reaction was quenched with brine, extracted three times with EtOAc, dried over MgSO4, and evaporated under reduced pressure, affording a crude extract (287 mg). Subsequently, the crude was applied to SiO_2_ column chromatography (hexane:EtOAc=1:1) affording **9** (133.5 mg) in a 58% yield (Scheme 2).

*Preparation of probe **3** from **9**:* To a solution of **6** (5 mg, 0.007 mmol) in THF (0.2 mL) were added 2,4,6-trichlorobenzoyl chloride (1.1 μL, 1.7 mg, 0.9 mol equiv. to **6**) and Et_3_N (2.14 μL, 1.56 mg, 2 mol equiv. to **6**), and the entire mixture was stirred at room temperature overnight (*ca.* 15 h). Then, the previously prepared **9** (5.1 mg, 0.016 mmol) was added, and the mixture was stirred for 1 h at room temperature. Next, DMAP (0.47 mg, 0.5 mol equiv. to **6**) was added, and an additional stirring was performed overnight at room temperature. The reaction mixture was directly evaporated under reduced pressure, affording a crude product (18.2 mg) that was applied to SiO_2_ column chromatography (CHCl_3_:CH_3_OH:H_2_O=30:3:1→15:3:1) giving **3** (2.6 mg) in a 35% yield (Scheme 2).

Compound **3**: a white powder, [α]_D_^25^ 145.2 (*c* 0.35, CHCl_3_), IR (KBr): 2934, 1735 (sh), 1699, 1613 cm^−1^, ^1^H NMR (CDCl_3_) δ: 7.80 (1*H*, d, *J*=8.2 Hz, 6″-H), 6.81 (1*H*, d, *J*=8.2 Hz, 5″-H), 6.71 (1*H*, s, 3″-H), 5.36 (0.75*H*, s, 5-Ha), 5.33 (0.25*H*, s, 5-Hb), 4.83 (0.75*H*, d, *J*=3.7 Hz, 12α-H), 4.49 (2.25*H*, m, 12β-H, 2″′-Ha, 2′-H), 4.32 (1*H*, m, 3′-H), 4.19 (2*H*, t, *J*=5.1 Hz, 7″-H), 4.14 (1*H*, m, 2″′-Hb), 3.91 (2*H*, t, *J*=5.1 Hz, 14″-H), 3.43–3.77 (14*H*, m, 8″, 9″, 10″, 11″, 12″, 13″, 1″′-H), 3.13 (1*H*, ddd, *J*=5.5, 6.4, 6.4 Hz, 1′-H), 2.90 (1*H*, dd, *J*=12.8, 4.6 Hz, 4′-Ha), 2.72 (1*H*, d, *J*=12.8 Hz, 4′-Hb), 2.61 (0.75*H*, m, 11-Ha), 2.45 (0.25*H*, m, 11-Hb), 2.37 (1*H*, ddd, *J*=3.7, 14.6, 14.6 Hz, 3α-H), 2.21 (2*H*, m, 8′-H), 2.02 (1*H*, brd, *J*=*ca.*14 Hz, 3β-H), 1.43 (0.75*H*, s, 14-Hb), 1.42 (2.25*H*, s, 14-Ha), 0.96 (0.75*H*, d, *J*=5.4 Hz, 15-Hb), 0.88 (2.25*H*, d, *J*=7.4 Hz, 13-Ha), 0.86 (0.75*H*, d, *J*=7.4 Hz, 13-Hb), 0.83 (2.25*H*, d, *J*=5.5 Hz, 15-Ha).

#### 1.2.3 Synthetic route to the biotinylated photoaffinity probe **4**

**Scheme 3:**
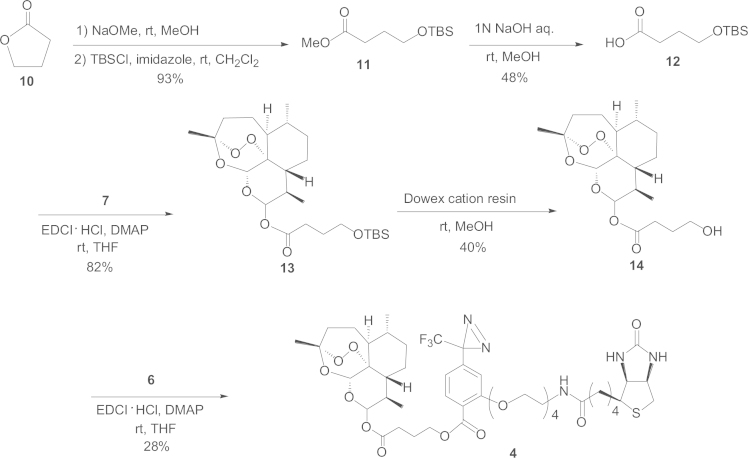
Synthetic route to the biotinylated photoaffinity probe **4**.

We began the process with γ-butyrolactone (**10**) that underwent methanolysis in the first step, followed by protection of the primary alcohol with *tert*-butyldimethylsilyl chloride (TBSCl) in dichloromethane in the second step, affording **11**, which was hydrolyzed under basic conditions to yield the *tert*-butyldimethylsilyl (TBS)-ether carboxylic acid **12**. Condensation of **12** with **7** under EDCI·HCl and DMAP in tetrahydrofuran (THF) led to **13**, which was deprotected using a Dowex cation resin (50WX8, 100-200 mesh, H Cation Exchange Resin, Sigma-Aldrich) in MeOH, affording **14**. Finally, condensation of **14** with **6** using EDCI·HCl and DMAP in THF afforded probe **4** in a 28% yield (Scheme 3).

*Preparation of **13** from **12**:* To a solution of **12** (19 mg, 0.087 mmol) in THF (1.2 mL) were added **7** (6.2 mg, 0.022 mmol), EDCI·HCl (31.68 mg, 2 mol equiv. to **12**), and DMAP (5.30 mg, 0.5 mol equiv. to **12**). The mixture was stirred at room temperature for 2 h. Then, EDCI·HCl (31.68 mg) and DMAP (5.30 mg) were added again, and an additional stirring was performed for 2 h. Following a reaction work up with brine, the mixture was extracted with EtOAc. The EtOAc layer was washed once with 5% HCl, once with saturated aqueous NaHCO_3_, once with brine, and then dried over MgSO4. Subsequent evaporation under reduced pressure produced **13** (18 mg) quantitatively (Scheme 3).

Compound **13**: a white powder, [α]_D_^25^ 148.3 (*c* 0.40, CHCl_3_), IR (KBr): 2928, 1750 cm^−1^, ^1^H NMR (CDCl_3_) δ: 5.81 (α, d, *J*=9.7 Hz, 12β-H), 5.44 (1*H*, s, 5-H), 3.89 (2*H*, t, *J*=6.4 Hz, 3′-H), 2.55 (1*H*, m, 11-H), 2.53 (1*H*, t, *J*=6.4 Hz, 1′-H), 2.34 (1*H*, ddd, *J*=3.7, 14.3, 14.4 Hz, 3α-H), 2.04 (1*H*, brd, *J*=*ca.*13 Hz, 3β-H), 1.43 (3*H*, s, 14-H), 0.96 (3*H*, d, *J*=6.1 Hz, 15-H), 0.84 (3*H*, d, *J*=7.4 Hz, 13-H).

*Preparation of probe **4** from **14**:* To a solution of **6** (0.7 mg, 0.001 mmol) in THF-CH_3_CN (1:1, 90 μL) were added the previously prepared **14** (0.8 mg, 0.002 mmol), EDCI·HCl (0.63 mg, 3 mol equiv. to **6**), and DMAP (0.07 mg, 0.5 mol equiv. to **6**), and then the mixture was stirred at room temperature for 3 h. Then, EDCI·HCl (0.63 mg) and DMAP (0.07 mg) were added, and an additional stirring was performed at room temperature for 30 min, followed by another stirring performed at 40 °C for 2 h. Subsequently, the reaction mixture was evaporated under reduced pressure, affording a crude product (4.2 mg) that was applied to SiO_2_ column chromatography (CHCl_3_:CH_3_OH:H_2_O=10:3:1→benzene: acetone=1:1 →1:2) giving **4** (0.3 mg) in a 28% yield (Scheme 3).

Compound **4**: a white powder, [α]_D_^25^ 158.8 (*c* 0.45, CHCl_3_), IR (KBr): 2930, 1735 (sh), 1698, 1608 cm^−1^, ^1^H NMR (CDCl_3_) δ: 7.79 (1*H*, d, *J*=8.5 Hz, 6″-H), 6.82 (1*H*, d, *J*=8.5 Hz, 5″-H), 6.71 (1*H*, s, 3″-H), 5.97 (1*H*, d, *J*=9.8 Hz, 12β-H), 5.49 (1*H*, s, 5-H), 4.94 (2*H*, t-like, *J*=*ca.* 5 Hz, 3″′-H), 4.51 (1*H*, m, 2′-H), 4.32 (1*H*, m, 3′-H), 4.20 (2*H*, m, 7″-H), 3.90 (2*H*, m, 14″-H), 3.42–3.76 (12*H*, m, 8″, 9″, 10″, 11″, 12″, 13″-H), 3.13 (1*H*, ddd, *J*=4.9, 7.3, 7.3 Hz, 1′-H), 2.92 (1*H*, dd, *J*=12.8, 4.9 Hz, 4′-Ha), 2.72 (2*H*, m, 1″′-Ha, 4′-Hb), 2.56 (1*H*, m, 11-H), 2.34 (2*H*, m, 3α-H, 1″′-Hb), 2.21 (2*H*, m, 8′-H), 2.02 (1*H*, brd, *J*=*ca.* 14 Hz, 3β-H), 1.41 (3*H*, s, 14-H), 0.98, 0.92 (both 3*H*, d, *J*=6.1, 7.3 Hz, 15-H, 13-H).

#### 1.2.4 Synthetic route to the biotinylated affinity probe **5**

**Scheme 4:**
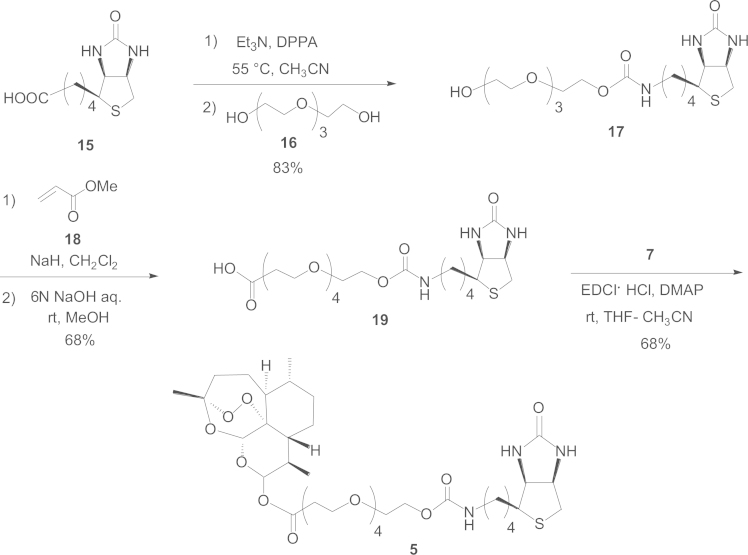
Synthetic route to the biotinylated affinity probe **5**.

We started with d-biotin (**15**) that underwent a Curtius rearrangement in the first step, followed by condensation with tetraethylene glycol (**16**) in the second step to afford **17**, which underwent a Michael addition to **18** and then hydrolyzed under basic conditions to afford the affinity labeling unit **19**. Condensation of **19** with **7** using EDCI·HCl and DMAP in THF-CH_3_CN=1:1 afforded probe **5** in a 68% yield (Scheme 4).

*Preparation of **19** from **17**:* To a solution of **17** (6.3 mg, 0.0145 mmol) in CH_2_Cl_2_ (144 μL) cooled to 0 °C were added NaH (588 μg, 1.0 mol equiv. to **17**) and **18** (6.5 μL, 6.2 mg, 5.0 mol equiv. to **17**). The reaction mixture was stirred at 0 °C for 1 h, which was then worked up with saturated aqueous NH_4_Cl until neutral pH. Following evaporation under reduced pressure, a crude product (22 mg) was obtained and subsequently applied to SiO_2_ column chromatography (CHCl_3_:CH_3_OH=18:1), affording the methyl ester derivative **19′** (6.7 mg) in an 88% yield. Then, in the second step, to a solution of **19′** (6.7 mg, 0.013 mmol) in MeOH (150 μL) was added 150 μL of 6 N aqueous NaOH. The reaction mixture was stirred at 40 °C for 15 min. Then, Dowex cation resin (50WX8, 100-200 mesh, H Cation Exchange Resin, Sigma-Aldrich) was added until neutral pH. Following filtration over a cotton pad, the mixture was evaporated under reduced pressure. The afforded crude product (6.9 mg) was applied to SiO_2_ column chromatography (CHCl_3_:CH_3_OH:H_2_O=15:3:1), giving **19** (4 mg) in a 68% yield (Scheme 4).

Compound **19**: a white powder, [α]_D_^25^ 24.2 (*c* 0.33, CHCl_3_), IR (KBr): 3296, 2928, 1703 cm^−1^, ^1^H NMR (CDCl_3_) δ: 4.51 (1*H*, m, 2-H), 4.34 (1*H*, m, 3-H), 4.23 (2*H*, t, *J*=4.9 Hz, 10′-H), 3.74 (2*H*, t, *J*=6.1 Hz, 2″-H), 3.61–3.70 (14*H*, m, 3′, 4′, 5′, 6′, 7′, 8′, 9′-H), 3.23 (1*H*, m, 1-H), 3.13 (2*H*, m, 8-H), 2.90 (1*H*, brd, *J*=*ca.*12 Hz, 4-Ha), 2.73 (1*H*, d, *J*=12.2 Hz, 4-Hb), 2.61 (2*H*, t, *J*=6.1 Hz, 1′-H).

*Preparation of the probe **5** from **19**:* To a solution of **19** (4 mg, 0.008 mmol) in THF-CH_3_CN (1:1, 500 μL) were added **7** (4.78 mg, 0.0168 mmol), EDCI·HCl (4.83 mg, 3 mol equiv. to **19**), and DMAP (0.52 mg, 0.5 mol equiv. to **19**), and then the mixture was stirred at room temperature for 6 h. Following subsequent evaporation under reduced pressure, the crude product (17.7 mg) was applied to SiO_2_ column chromatography (CHCl_3_:CH_3_OH:H_2_O=10:3:1→benzene:acetone=1:2→100% MeOH), giving **5** (4.3 mg) in a 68% yield (Scheme 4).

Compound **5**: a white powder, [α]_D_^25^ 164.9 (*c* 0.46, CHCl_3_), IR (KBr): 2928, 1740 (sh), 1703 cm^−1^, ^1^H NMR (CDCl_3_) δ: 5.79 (1*H*, d, *J*=9.8 Hz, 12β-H), 5.44 (1*H*, s, 5-H), 4.51 (1*H*, dd, *J*=7.7, 5.1 Hz, 2′-H), 4.35 (1*H*, dd, *J*=7.7, 4.6 Hz, 3′-H), 4.20 (2*H*, m, 10″-H), 3.76 (2*H*, dd, *J*=6.6, 6.5 Hz, 2″-H), 3.62–3.69 (14*H*, m, 3″, 4″, 5″, 6″, 7″, 8″, 9″-H), 3.15 (3*H*, m, 1′, 8′-H), 2.90 (1*H*, dd, *J*=4.6, 12.6 Hz, 4′-Ha), 2.74 (1*H*, d, *J*=12.6 Hz, 4′-Hb), 2.69 (2*H*, dt, *J*=12.8, 6.6 Hz, 1″-Ha), 2.60 (2*H*, dt, *J*=12.8, 6.5 Hz, 1″-Hb), 2.55 (1*H*, m, 11-H), 2.37 (1*H*, dt, *J*=3.9, 13.7 Hz, 3α-H), 2.03 (1*H*, brd, *J*=*ca.*14 Hz, 3β-H), 1.43 (3*H*, s, 14-H), 0.96 (3*H*, d, *J*=5.9 Hz, 15-H), 0.84 (3*H*, d, *J*=7.1 Hz, 13-H). FAB-MS *m/z*: 774 [M+H]^+^.

## Conflict of interest

The author has no conflicting interest to declare.
